# Measles outbreak linked to insufficient vaccination coverage in Nouvelle-Aquitaine Region, France, October 2017 to July 2018

**DOI:** 10.2807/1560-7917.ES.2018.23.30.1800373

**Published:** 2018-07-26

**Authors:** Anne Bernadou, Cyril Astrugue, Matthieu Méchain, Valérie Le Galliard, Catherine Verdun-Esquer, France Dupuy, Julia Dina, Fatima Aït-Belghiti, Denise Antona, Stéphanie Vandentorren

**Affiliations:** 1 Santé Publique France, French National Public Health Agency, Regional office in Nouvelle-Aquitaine, Bordeaux, France.; 2 European Programme for Intervention Epidemiology Training (EPIET), European Centre for Disease Prevention and Control (ECDC), Stockholm, Sweden; 3 Regional health agency in Nouvelle-Aquitaine, Bordeaux, France; 4 Occupational medicine service of Bordeaux University hospital, Bordeaux, France; 5 Student health service, Bordeaux, France; 6 National Reference Center for Measles, Mumps and Rubella, CHU de Caen, Virology Department, Caen, France; 7 Santé publique France, French National Public Health Agency, Infectious Diseases Department, Saint-Maurice, France

**Keywords:** measles, measles-mumps-rubella (MMR) vaccine, outbreaks, Air-borne infections, Students, Healthcare facilities

## Abstract

On 30 October 2017, an outbreak of measles started in the Nouvelle-Aquitaine (NA) region in France among Bordeaux University students before spreading to other regions. Until 1 July 2018, 1,101 cases were reported in NA, including 98 complications and two deaths. Cases were related to clusters (e.g. students, healthcare workers) in 16%; 81% of cases were not vaccinated against measles as recommended. Vaccination coverage above herd immunity threshold remains the main preventative outbreak measure.

On 30 October 2017, a measles outbreak started among students attending Bordeaux University in the southwest of France. It rapidly spread into the general population of Bordeaux’s Gironde district, into Nouvelle-Aquitaine region (NA) and then onto other regions. We conducted an investigation to describe the outbreak, identify clusters and stop further transmission.

## Case definitions

In France, measles became a notifiable disease in 2005 to improve the detection of active transmission and increase the specificity of the diagnosis through laboratory confirmation of clinical cases. Clinicians and microbiologists are requested to report suspected and confirmed measles cases immediately to the Regional Health Agencies (ARS) responsible for implementing control measures.

We included all cases reported in the mandatory notification system (possible, probable and confirmed) who lived in NA and developed rash onset between 30 October 2017 and 1 July 2018 (Box). We interviewed cases to identify contacts at risk and detect clusters in high risk groups.

Box **Definition of cases and other categories followed-up during the measles outbreak, Nouvelle-Aquitaine region, France, 30 October 2017–1 July 2018**

**Measles cases**

Possible case

Person presenting with fever ≥ 38.5 °C, maculopapular rash and at least one of the following symptoms: conjunctivitis, coryza, cough or Koplik’s spots

Probable case

Person meeting the clinical criteria and with an epidemiological link to a laboratory-confirmed case

Confirmed case

Person meeting the clinical criteria and with laboratory-confirmed measles infection detected by serology or RT-PCR in serum or oral fluid and not recently vaccinated

**Other categories**

Cluster

At least three cases including one confirmed case in high-risk groups

High-risk groups

Groups with high number of susceptibles and/or people at risk to develop complications

## Outbreak description

**Figure 1:**
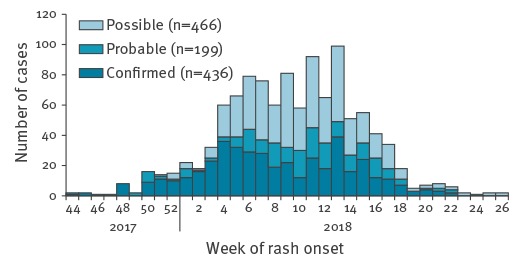
Notified measles cases by week of rash onset, Nouvelle-Aquitaine region, France, 30 October 2017–1 July 2018 (n = 1,101)

Source: Notification data-Santé publique France.

In total, 1,101 cases (466 possible, 199 probable, 436 confirmed) were reported in NA which represented 41% of the cases reported in France. Following the start of the measles outbreak on 30 October 2017, cases increased from week 50 2017 and peaked during week 13 2018 (Figure 1). Five hundred and eighty-one cases (53%) were male, and 646 (59%) cases were older than 15 years. The highest cumulative incidence rate was observed in children < 1 year of age (160.8/100,000). The incidence rate was 40.7 per 100,000 in children of 1–14 years, 41.7 in young adults of 15–29 years and 4.1 in adult’s ≥ 30 years. Of the 1,101 cases, 248 cases (23%) were hospitalised, 98 cases (9%) had complications and two died – one of respiratory complications (in their mid-30s, with underlying risk factors: morbid obesity and heavy smoking) and the second of neurologic complications (teenager, with an immunodeficiency).

**Figure 2:**
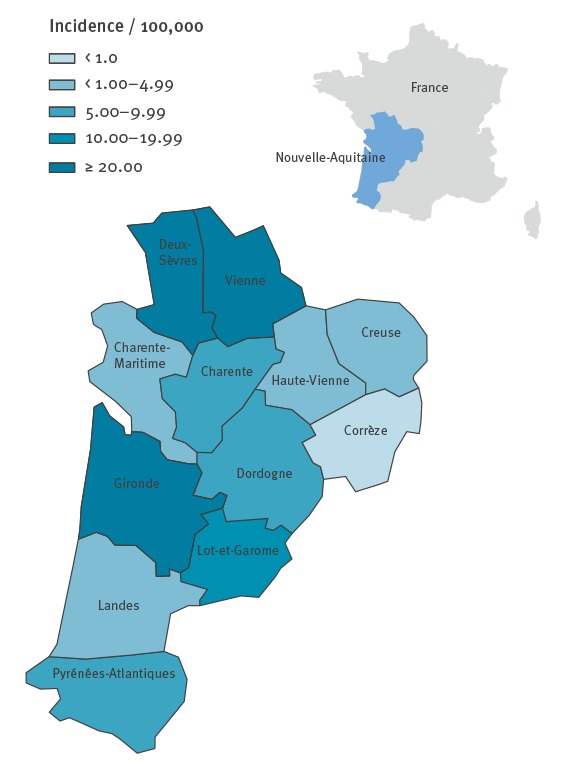
Crude incidence rate of measles, by district, Nouvelle-Aquitaine region, France, 30 October 2017–1 July 2018 (n = 1,101)

Source: Notification data-Santé publique France, extracted on 23 July 2018.

The majority of the 1,101 cases (n = 617; 56%) lived in Gironde, which is NA’s biggest district; all districts reported cases (Figure 2). In NA, the cumulative crude incidence rate was 18.5 per 100,000 population (vs 4.0/100,000 in France during the same period), and reached 39.4 per 100,000 population in Gironde.

Number of cases: Charente (n = 18), Charente-Maritime (n = 25), Corrèze (n = 2), Creuse (n = 3), Deux-Sèvres (n = 89), Dordogne (n = 31), Gironde (n = 617), Haute-Vienne (n = 14), Landes (n = 19), Lot-et-Garonne (n = 41), Pyrénées-Atlantiques (n = 39), Vienne (n = 203).

A total of 594 (69%) of 860 cases with a known vaccination status were unvaccinated, 128 (15%) had received one dose, 118 (14%) two doses and 20 (2%) had been vaccinated with an unspecified number of doses.

Between 30 October 2017 and 1 July 2018 (study period) genotyping was conducted on 125 specimens by the French National Reference Laboratory for Measles in Caen, leading to the identification of genotype D8 in 105 cases (84%) and genotype B3 in 12 cases (10%). For six specimens the sequencing test was in failure due to limited material.

A total of 176 cases (16%) were related to clusters (n = 19) (Table 1).

**Table 1:** Characteristics of clusters identified in Nouvelle-Aquitaine region, France, 30 October 2017–1 July 2018, (n = 176)

Clusters	Number of cases	Week of rash onset of the first case	Number of weeks between the first and the last case	Median age (range)	Number of non-vaccinated^a^		Number of hospitalisations
					N	%	
**Hospital**							
University of Bordeaux^b^	40	46–2017	19	20 (17–31)	33	100	8
University of Poitiers	21	09–2018	7	20 (18–55)	11	65	2
**Healthcare facilities**							
Hospital in Bordeaux^c^	26	48–2017	20	26 (0–72)	10	62	7
Hospital in Poitiers	6	05–2017	1	31.5 (28–36)	4	80	2
**Place assigned for France’s nomadic minorities**							
Area 1 in Gironde	8	52–2017	12	18.5 (1–29)	7	100	4
Area 2 in Gironde	3	06–2018	2	3 (1–21)	3	100	1
Area 3 in Gironde	10	08–2018	7	20 (0–28)	5	83	2
Area 4 in Gironde	3	07–2018	3	10 (0–26)	3	100	0
Area 1 in Vienne^b^	9	03–2018	3	16 (1–49)	5	100	7
Area 2 in Vienne	7	04–2018	4	29 (10–35)	2	100	3
Area 1 in Deux-Sèvres	3	05–2018	4	7 (2–8)	NA	NA	2
Area 2 in Deux-Sèvres^b^	6	07–2018	6	2 (1–24)	5	100	0
Area 3 in Deux-Sèvres	3	04–2018	2	2 (0–6)	2	100	0
**Private school**							
Private school of Bordeaux	5	01–2018	1	5 (4–8)	5	100	0
Private school of Vienne^d^	9	11–2018	3	5 (1–38)	9	100	1
Private school of Deux-Sèvres	5	12–2018	5	11 (7–34)	4	80	1
**Socially vulnerable people**							
Migrants	3	12–2018	2	27 (26–45)	2	100	1
Prisoners	6	13–2018	6	36 (30–43)	2	100	0
**Childcare centre**							
Childcare centre in Bordeaux	3	09–2018	6	0 (0–1)	3	100	1

NA: not available.

Source: Santé publique France.

^a^ Number of unvaccinated or vaccinated with one dose MMR among with a known vaccination status.

^b^ One epidemiologically linked case lived in another region (not included).

^c^ Vaccination coverage survey conducted among medical staff in Bordeaux University Hospital during the outbreak (in units with high-risk of transmission as emergency, paediatrics, infectious diseases, etc.) estimated a vaccination coverage of 14% for one MMR dose and 34% for two MMR doses.

^d^ Eight epidemiologically linked cases lived in another region (not included).

## French vaccination schedule

Measles-containing vaccines have been included in the French vaccination schedule since 1983 with the first dose administered at 12 months of age. In 1996, a second dose was introduced and initially administered at 11 years of age, but changed to 3 to 6 years of age in 1997. This led to a decrease in the number of estimated measles cases (clinical case definition) from 331,000 cases in 1986 to 4,448 cases in 2004 [1]. Despite its increase, vaccination coverage in 2000 remained under the target of 85% for the first dose. A large outbreak occurred between 2008 and 2011, leading to more than 22,000 reported cases [2]. Since 2005, two doses of measles, mumps and rubella (MMR) vaccine has been recommended in France; the first dose administered at 12 months of age and the second dose between 16 and 18 months. A two-dose catch-up measles vaccination is recommended for people born in 1980 and after [3].

## Control measures

Specific control measures targeting populations affected by the outbreak were implemented by ARS according to national guidelines, including catch-up and post-exposure vaccination and/or prophylaxis with immunoglobulin for people at high-risk of severe disease [4].

Since December 2017, information regarding the recommended vaccination schedule has been disseminated to the population in NA via various means e.g. newspapers and social media, as well as to health workers (HCWs) and specific at-risk populations such as France nomadic minorities.

Infection control measures such as isolation of cases and airborne precautions were implemented in healthcare facilities and susceptible individuals (unvaccinated HCWs and patients’ contacts without history of measles) were vaccinated to reach two doses of MMR vaccine. MMR catch-up campaigns were undertaken targeting the population around several clusters: University students (Bordeaux and Poitiers), HCWs in Bordeaux hospital, places of France’s nomadic minorities in Gironde and Vienne districts and the prison in Vienne. The outbreak control team established mobile vaccination teams to increase vaccination uptake among these groups.

## Discussion

Since May 2017, there has been an increase in measles incidence in several Europe countries [5]. France has been experiencing a large measles outbreak that started in NA, rapidly spread to other regions and resulted in a total of 2,663 cases in France between 30 October 2017 to 1 July 2018 [6]; among which 1,101 occurred in NA (41%) where clusters among under-vaccinated groups were documented.

We also observed cases among fully vaccinated people and the proportion of fully vaccinated among cases was higher than what was observed in France during the 2008–11 epidemic (14% vs 3–4%, respectively). This may be linked to the waning immunity over time among vaccinated people and to a higher proportion of vaccinated cases in a population where the level of vaccination coverage becomes optimal [2,7,8]. But this finding should nevertheless be further investigated.

The second MMR dose vaccination coverage administered at 2 years of age increased from 45–64% in 2010 to 71–81% in 2015 among the districts of NA; yet it remained insufficient to reach the herd immunity threshold of 95% to achieve a high level of population immunity and measles elimination [9,10]. Vaccination coverage is most likely lower among minority groups such as nomadic minorities or confessional groups, but it would not alone account for the low vaccination coverage of the region; further studies are ongoing to estimate vaccination coverage among France's nomadic minorities.

The main circulating genotypes reported in NA were B3 and D8, as observed in the rest of the country and Europe [11].

Two clusters started among university students, which belong to large cohorts with increasing numbers of under-vaccinated (susceptible) people. Crowded environments with close-contact situations such as those observed in students’ communities can greatly contribute to the emergence of outbreaks. Moreover complications are often more frequent and more severe for these age groups [12].

Two of the clusters occurred in healthcare facilities involving HCWs and hospitalised patients, also described in others recent outbreaks [2,13,14]. Despite the existing recommendations for HCWs to be vaccinated against measles, we found among clusters in NA that vaccination coverage of HCWs remained insufficient. Systematic checks and updating of the vaccination status of HCWs and implementation of other control measures are needed to avoid nosocomial measles transmissions, which may have been a main transmission route in this outbreak.

The high proportion of hospitalised cases could suggest that severe cases are more likely to be reported than less severe cases diagnosed by general practitioners (GPs). During the 2008–11 epidemic, a cross-sectional survey conducted among blood donors 18–32 years old in the south-east of France found that only 45% of cases had been notified [15]. Further, during the first weeks of this outbreak, investigations indicated that some of the GPs waited for a laboratory confirmation before notifying the cases. This delay in reporting might have led to a delay in the implementation of control measures and further transmission of disease.

## Conclusion

This measles outbreak was the consequence of a suboptimal vaccination coverage in children and insufficient catch-up vaccinations in young adults, resulting in a large reservoir of susceptible individuals. There is an urgent need to improve vaccination coverage with two doses of MMR in NA and France, especially among young adults and in hard-to-reach populations. In 2018, a new law has been enacted that makes MMR vaccination mandatory for the new birth cohorts, which should help reaching the vaccination target of 95% in the future [16]. Prevention of measles transmission in healthcare settings should be strengthened by implementing effective infection control practices and ensuring that all HCWs are immune against measles.
